# Basin-Scale Pollution Loads Analyzed Based on Coupled Empirical Models and Numerical Models

**DOI:** 10.3390/ijerph182312481

**Published:** 2021-11-26

**Authors:** Man Zhang, Xiaolong Chen, Shuihua Yang, Zhen Song, Yonggui Wang, Qing Yu

**Affiliations:** 1 School of Resource and Environmental Sciences, Wuhan University, Wuhan 430079, China; 2019202050070@whu.edu.cn; 2 Beijing Tsinghua Holdings Human Settlements Environment Institute, Beijing 100083, China; cxl@bjenv.com; 3 Yanshan Experimental High School, Yanshan 663100, China; yangshuihua@163.com; 4 Key Laboratory of Regional Ecology and Environmental Change, School of Geography and Information Engineering, China University of Geosciences, Wuhan 430074, China; songzhen79@163.com (Z.S.); yuqing0334@163.com (Q.Y.)

**Keywords:** basin-scale pollution loads, source apportionment, pollution source analysis framework, coupled models, SWAT model, Tuojiang River basin

## Abstract

Pollutant source apportionment is of great significance for water environmental protection. However, it is still challenging to accurately quantify pollutant loads at basin-scale. Refined analytical methods combined the pollution discharge coefficient method (PDCM), field observation, and numerical model (Soil & Water Assessment Tool, SWAT) to make quantitative source appointment in the Tuojiang River, a key tributary of the upper Yangtze River. The chemical oxygen demand (COD), total nitrogen (TN), total phosphorus (TP), and ammonia nitrogen (N-NH4^+^) were analyzed. Results showed that the urban sewage treatment plant point source has the largest contribution to COD, TN, and N-NH4^+^, while TP is mostly from the agricultural sources throughout the year. The total inflowing loads of pollution sources are significantly affected by rainfall. The overall pollution characteristics showed that pollutant loads present in different seasons are as follows: wet season > normal season > dry season. The month with the highest levels of pollutants is July in the wet season. Among the nine cities, the city that contributes the most COD, TN and N-NH4^+^, is Neijiang, accounting for about 25%, and the city that contributes the most TP is Deyang, accounting for 23%. Among the sub-basins, the Fuxi River subbasin and Qiuxihe River subbasin contribute the most pollutant loads. The technical framework adopted in this paper can be used to accurately identify the types, administrative regions and sub-basins of the main pollution sources in the watershed, which is conducive to management and governance of the environment.

## 1. Introduction

Water pollution is one of the most serious water environmental problems faced by many countries and cities. Exploring the topic of responsibility in water pollution is the foundation of ecological environmental control and protection [1]. Watershed water ecological environmental control and protection should be based on the source apportionment and control [2]. However, with the rapid development of the social economy, especially the industrial development and urban expansion, water pollutant sources become more diverse and complex [3,4,5].

The process of contaminants entering the river is characterized by complex mechanisms with irregular occurrence [6]. Point sources (PS) usually have fixed outfalls from industrial sewage, urban sewage treatment plants, the large-scale livestock and poultry breeding industry, etc. The inflow flux of point sources could be calculated based on monitoring data for the point sources with outlets directly entering the river [7]. However, difficulties in streamflow loss make PS that outlets with a certain distance from the river network more complicated. Non-point sources (NPS) pollution caused by intensive agricultural planting, livestock and free range poultry and aquaculture greatly contributed to declining water quality and aquatic ecosystems, which is characterized by dispersive, random and uncertain pollution discharge compared with PS [6]. Complexities in monitoring and controlling makes NPS pollution more complicated than point source pollution [8]. Pollutant sources apportionment based on the response of water quality to pollutant discharge is one of the main research methods in the river basin. Many methods have been put forward for sources apportionment recently, such as inventory analysis methods [9,10], statistical methods [11,12,13], empirical models [14], and mechanism models [15,16,17]. The numerical model methods largely based on relatively simple mathematical calculation methods have the problem of over-generalization and unable to describe pollution spatial distribution. On the one hand, the estimation accuracy of the above-mentioned method depends on a large number of parameters, requirements for a large body of input data, leading to difficulties in calibrating and validating these models [18,19]. On the other hand, complex factors such as land-use type and pollutants infiltration loss does not fully take into account in the process of pollutants discharging into the river. Summing up, to carry out refined water environment management, it is necessary to establish a new analytical framework of pollution sources based on the characteristics of pollution discharge and inflow into the river from PS and NPS. The target is more explicitly displayed in the characteristics of discharge and distributed the load from different pollution sources.

The Yangtze River, the longest river in Asia, flows for more than 6300 km and is characterized by complex and indistinguishable pollution sources, with water quality significantly different at the spatial scale, similar to the Mississippi River Basin and the Ganges River Basin. In 2016, the Yangtze River Economic Belt (YREB) was launched as a national strategy. The YREB region has been facing increasingly severe water ecological environmental pressure due to high energy consumption and fast-growing urbanization [20,21]. The Tuojiang River Basin is an important tributary affecting the water quantity and quality of the Yangtze River [22]. The local government has made great efforts to control pollution sources, but pollution in the river basin remains severe [23,24]. The main reason is that the basin-scale pollution loads have not been analyzed, which leads to the lack of pertinence of the methods adopted. Therefore, it is urgent to quantize the pollution sources loads for maintaining the sustainable ecological function of the Tuojiang River and the Yangtze River.

Combining the above analysis, fully considering the feature of pollutants, this study aims to: (1) propose a set of pollution source analysis technology based on the characteristics of different pollution sources. (2) calculate the load of PS directly inflow into the river using investigating and monitoring. (3) simulate the discharge quantity of NPS and PS that indirect inflow into the river with coupled models. (4) finely analyze the inflowing loads of different pollution sources and their spatial and temporal distribution characteristic. The methods used in this paper could decrease the uncertainty of pollution sources resolution and enhance the accuracy of calculating the number of pollutants discharging into the river.

## 2. Data and Methods

### 2.1. Study Areas and Data Sources

**Figure 1:**
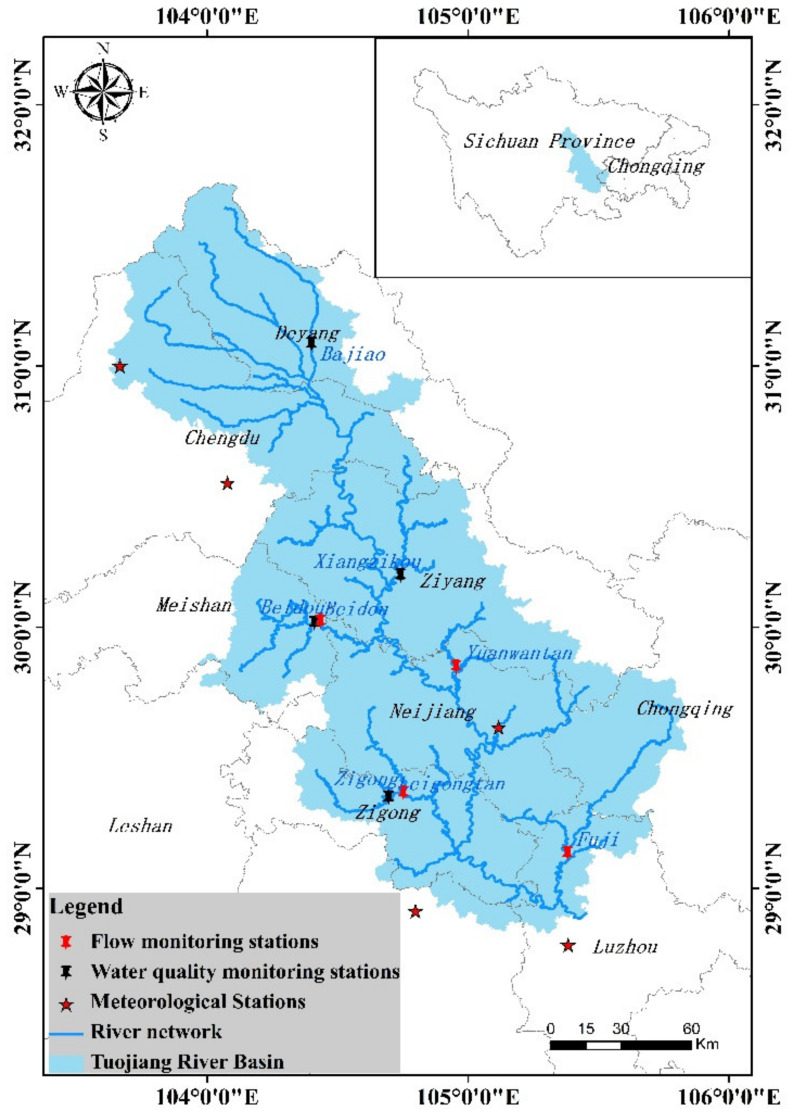
Geographical location, regions, river networks, and the locations of the flow and water quality monitoring stations of the Tuojiang River Basin.

As shown in Figure 1, the Tuojiang River lies in the upper reaches of the Yangtze River and the center of Sichuan Province, with length of 627.4 km and area of 25,576 km^2^. The average annual precipitation in the basin is 1200 mm, and the flow rate is 35.1 billion cubic meters [22]. The river basin covers Chengdu, Deyang, Leshan, Luzhou, Meishan, Neijiang, Yibin, Ziyang, Zigong, involving a total of nine cities and 36 counties (cities and districts), including 567 townships. Tuojiang River is the river with the largest concentration of industrial cities in Sichuan Province. The water environment of the Tuojiang River Basin is affected by many factors and is a typical representative of the compound pollution of the water environment in China [25].

In this study, the chemical oxygen demand (COD), total nitrogen (TN), total phosphorus (TP), and ammonia nitrogen (N-NH4^+^) were chosen as indicators of pollution source analysis. The data of NPS were calculated based on the statistical yearbook of cities in Sichuan Province (2016). The pollution source data of point source were mainly from the environmental statistics of Sichuan Province (2016), as well as the hydrological and water quality monitoring data.

### 2.2. Methods

**Figure 2:**
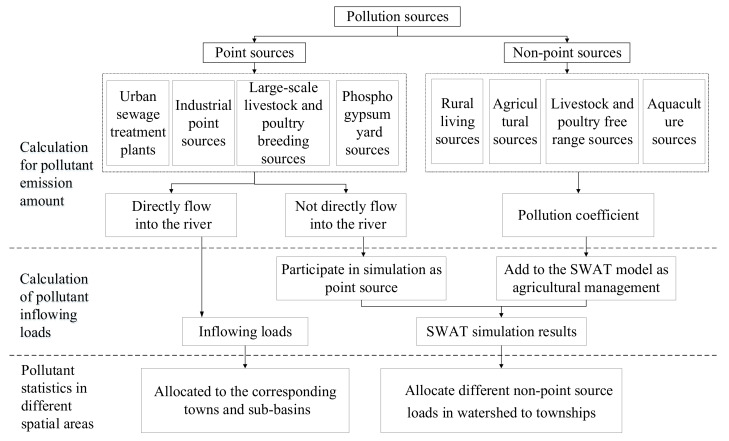
The framework of basin-scale source apportionment, “Directly flow into the river” refers to the Tuojiang main stream.

Since there are obvious differences between the PS and NPS, such as the pollutants produced and the approach of pollutants inflow into the river, pollution sources apportionment were divided into the pollutants emission calculation process and the contaminants loading simulation process. For the point sources that directly flow into the main stream of the Tuojiang River, we used the obtained point sources data to directly distribute to the corresponding subbasin. The framework of basin-scale source apportionment is shown in Figure 2.

#### 2.2.1. Calculation for Pollutant Emission Amount

PS is mainly derived from industrial sewage, urban sewage treatment plant sewage [7], large-scale livestock, and poultry breeding industry [26]. The phosphogypsum yard was one of the main industrial sources of pollution in the upper reaches of the Tuojiang River Basin. The phosphogypsum yard commonly has a separate sewage outlet, as one of a single PS in this study. Based on the discharge characteristics of the PS and the investigation and survey in Tuojiang River Basin, PS was divided into four categories: urban sewage treatment plant, industrial sewage, large-scale livestock and poultry breeding industry source, and phosphogypsum yard source. The emission amount was calculated using the field survey and monitoring method [27].

To quantify the NPS pollution load precisely, NPS sources in this study were divided into rural living sources, agricultural sources, livestock and poultry free range sources, and aquaculture sources.

(1) In rural areas, domestic sewage and garbage produced from residents’ daily life were discharged randomly due to imperfect sewage collection channels, and the drainage facilities were not improved [2]. Combined the statistical yearbook of cities in Sichuan Province (2016), the quantity of rural resident population and the daily pollution discharge coefficient of population were used to calculate the annual emissions in the study.
(2) Agricultural planting pollution caused by excessive chemical fertilizers in agricultural producing activities, of which the annual discharge was related to the pure consumption of nitrogen and phosphate fertilizers. In this paper, the amount of fertilizer applied and the loss coefficient of nitrogen and phosphorus fertilizers were used to calculate the pollutant emissions from agricultural planting [28,29].
(3) Livestock and free range poultry sources refer to the pollution load produced by livestock and poultry that have not formed large-scale breeding. The calculation of pollutant emission amount takes into account the total number of free range livestock and poultry and the daily discharge coefficient of livestock and poultry pollutants which derived from the “Handbook of Pollution Production and Emission Coefficients for the First National General Survey of Pollution Sources”, result of the first pollution sources census of China.
(4) Aquaculture pollution involves two types, one is the excrement of fishing pollutant, and the other is excessive fishing fodder feeding. Given the characteristics of aquaculture pollutants, the annual discharge was calculated by bait coefficient, content percentage of nitrogen and phosphorus in fodders and cultured fishery products respectively.

The specific formulas of the four pollution sources in this study are shown in Table 1.

Emission factors of these four pollutant sources were obtained from the first and second pollution sources census of China (Table 2), where the basic situation of various pollution sources, the amount of major pollutants discharged, and the situation of pollution control can been found [1].

#### 2.2.2. Simulation of Pollutant Inflowing Loads

Pollutants discharge process is affected by many factors such as terrain, vegetation, land-use type, human activities, etc. [30], which lead to differences between the pollutant emission amount and the pollutant inflowing loads. Therefore, it is indispensable to calculate the total pollutant load flowing into the river, i.e., the inflowing load. The calculation of point source pollutant inflowing loads is divided into two parts. For the point source directly discharged into the river, the field survey and monitoring method were adopted. Its emissions are equivalent to inflowing loads, so this aspect of point sources is not included in the SWAT model simulation. For the other point sources with a certain distance from the river, the geographical location of the outlets and the target river of the point source were first determined through field investigation. Considering that discharge characteristics were similar to non-point source pollutants, the additional point sources and were treated the same as the non-point source pollutants in the calculation process.

The SWAT model was largely used to simulate the spatiotemporal distribution and source attribution of pollutant loads [31,32] and assess the impacts of management measures on nutrient export rates [33,34], i.e., which hydrologic cycle simulation was based on a water balance equation and used the Green-Ampt method or the Soil Conservation Service (SCS) curve number (CN) technique [35] to calculate surface runoff volume. The SWAT model, however, did not specifically create a systematic solution for file preparation of point source and non-point source. Therefore, it can be necessary to perfect the file module field of SWAT model according to Chinese actual environmental conditions [36].

The special treatment of point source and non-point source was considered in many ways. First, by completing the construction of the SWAT model. Second, excluding point sources that directly flow into the river, they can be directly counted without simulation. Third, determining the subbasin id of the additional point sources outlets based on geographical location, and importing it into the model as a SWAT point source file. Finally, the above results of four NPS pollutant emission amount are added to the SWAT model as agricultural management to simulate non-point source discharge into the river. In the SWAT model, the carbon biological oxygen demand (CBOD) was simulated rather than the chemical oxygen demand (COD). We calculate the concentration of COD (*C_cod_*) according to the concentration of CBOD (*C_cbod_*), with correlation formula between them from monitoring data:

$$C_{cod}=2.0162\times C_{cbod}+10.583$$

#### 2.2.3. Pollutant Statistics in Different Spatial Areas

To provide an accurate scientific basis for the targeted environmental management, the SWAT simulation results and the monitored point sources were further distributed to different towns and sub-basins in the basin.

The monitored point source inflowing loads were directly allocated to the corresponding towns and sub-basins according to the location of its sewage outlet. For the SWAT simulation results, the contribution of different pollution sources to each subbasin is determined based on the simulation results of pollution load of each subbasin and the ratio between different point sources and non-point sources input files, and then determine the inflowing loads contribution of different spatial areas based on the relationship between the sub-basin and the township.

### 2.3. Model Development and Validation

#### 2.3.1. Input Data

The SWAT model was used to simulate streamflow and nutrient pollution in the Tuojiang River Basin, and multiple sources of data were required for the modeling (Table 3).

The 30 m digital elevation model (DEM) data were obtained from the Chinese Academy of Sciences mirror site. The meteorological data were obtained from the China Meteorological Science Data Center. A land-use type map was derived from the Sichuan Academy of Environmental Sciences. The soil data were derived from the 1 km × 1 km Harmonized World Soil Database (HWSD). To calibrate and validate the model, water quality monitoring data were collected from the Sichuan Provincial Ecological Environment Monitoring Station.

#### 2.3.2. Subbasin and Hydrological Response Unit Division

In this study, the tool ArcGIS 10.2 software (GeoScene Information Technology Co Ltd., Beijing, China) was used to perform the spatial analysis of this study based on the digital elevation model (DEM). The Tuojiang River Basin was delineated into 1487 sub-basins according to the actual situation and simulation accuracy requirements. Based on the sub-basins, the plots with similar or identical land-use, soil types and slope characteristics are divided into the same type of hydrological response unit (HRUs).

#### 2.3.3. Model Calibration and Validation

Calibration and validation were carried out to assess the performance of the model using several indices, including the SUFI-2 method, Nash-Sutcliffe efficiency (NSE) [35] and the coefficient of determination (R^2^) [37,38], in which runoff was used for hydrological verification, COD, N-NH4^+^, TN and TP concentrations were applied for water quality verification. When calibrating the streamflow and water quality, 2014 was used as the warm-up period, and 2015 and 2016 were used as the calibration and validation periods, respectively.

For hydrological simulation, R^2^ describes the proportion of the total variance in the measured data explained by the model. It ranges from 0 to 1.0, with higher values indicating less error variance, and values greater than 0.50 are considered acceptable [39], the NSE value between 0.36 and 0.75 is considered satisfactory, and values below 0.36 is considered unsatisfactory [35].

**Figure 3:**
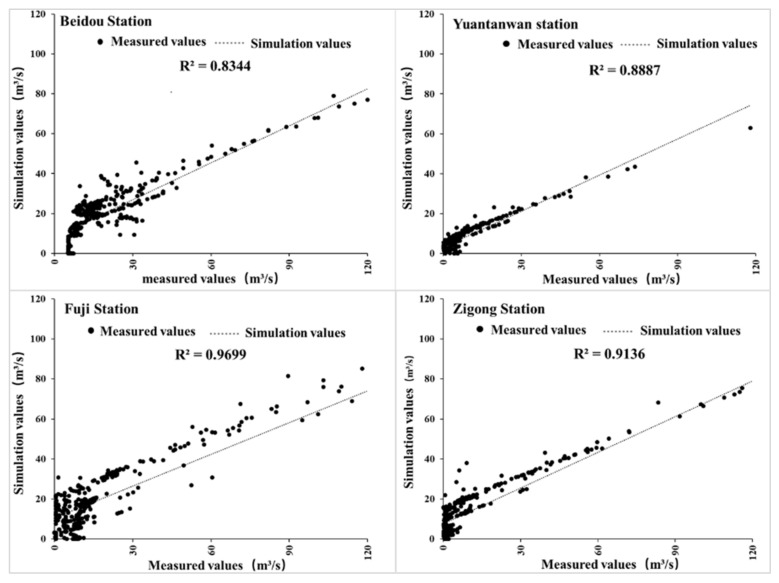
Comparison between simulated value and measured values of Beidou, Yuanwantan, Fuji, Zigong hydrological station in 2016.

Figure 3 shows the observed and simulated daily streamflow at the Beidou, Yuanwantan, Fuji, Zigong hydrological station during 2016, respectively. The R^2^ values of the four flow monitoring stations were 0.8344, 0.8887, 0.9699 and 0.9136 respectively, which were far beyond the acceptable range of 0.50 and achieved a very good simulation effect. The quantity range and change trend of streamflow have good consistency between model calculation results and the observed data from Figure 3.

**Figure 4:**
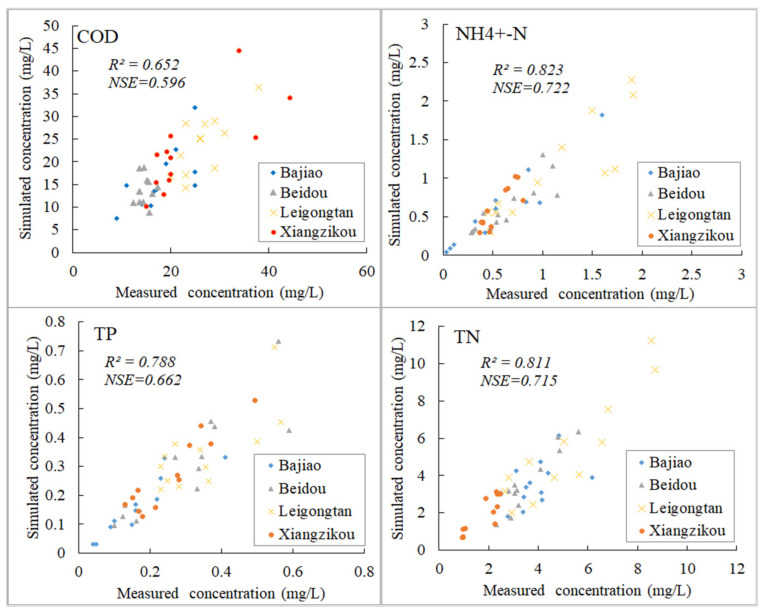
Monthly simulation results and measured data of COD, N-NH4^+^, TN, TP in Xiangzikou, Bajiao, Beidou, Leigongtan, with R^2^ and NSE of water quality verification.

The R^2^ and NSE of water quality verification (COD, N-NH4^+^, TN, TP) of 2016 are shown in Figure 4. The R^2^ values of the four water quality monitoring stations (Bajiao, Beidou, Leigongtan and Xiangzikou) were in the range 0.652~0.823, and the NSE values were in the range 0.596~0.722, which are all satisfactory. Compared to the other three indicators, the simulation result of COD concentration was the worst, but the overall results reached an acceptable level. The Bajiao station of the four stations has a poorer simulation effect than the other three because the station is located on the main stream of the Tuojiang River, and the simulation effect of SWAT for large river channels needs to be improved [40]. The simulation results of the other stations located in the tributaries are all satisfactory and can reflect the process of pollutants entering the river. As a whole, the hydrological and water quality responses were effectively calibrated and the model can be applied to the simulation of the Tuojiang River Basin.

## 3. Results and Discussion

### 3.1. Total Pollution Emissions

According to the numerical calculation results, we calculated that the total pollutants of COD, TN, TP, and N-NH4^+^ were 545,000 tons, 106,100 tons, 24,800 tons, and 60,900 tons respectively, as shown in Table 4.

As can be seen from Table 4, NPS total emissions more than PS total emissions. Urban sewage treatment plant and rural living source are the main pollutant sources of COD, TN, and N-NH4^+^. For TP, the agricultural source has the largest proportion, with a percentage of 67.74%, followed by urban sewage treatment plant and rural living source, with percentages of 14.11% and 10.48% respectively.

Presently, domestic sewage has become one of the most important water pollution sources in China [41]. The population density of Tuojiang River basin is much higher than that of other river basins [23], so the sewage load generated in the life of urban residents is relatively large. In addition, part of the industrial sewage would be processed by the urban sewage treatment plant before discharge. The above factors lead to the urban sewage treatment plant accounting for the largest proportion of the total pollution load of COD, TN, and N-NH4^+^.

The total population of the Tuojiang River Basin is 26.72 million, of which 15.15 million are agricultural population. Due to the continuous improvement of the level of agricultural development which has resulted in the bad situation that nitrogen, phosphorus, pesticides and other organic or inorganic pollutant entering the surface water, groundwater, and soil environments through surface runoff. Therefore, the agricultural pollution load generated in the basin is also relatively high. All these pollutions endanger the environment and the health of residents [42].

### 3.2. Temporal Characteristics of Pollution Loads

#### 3.2.1. Yearly Pollution Loads of Different Pollution Sources

**Figure 5:**
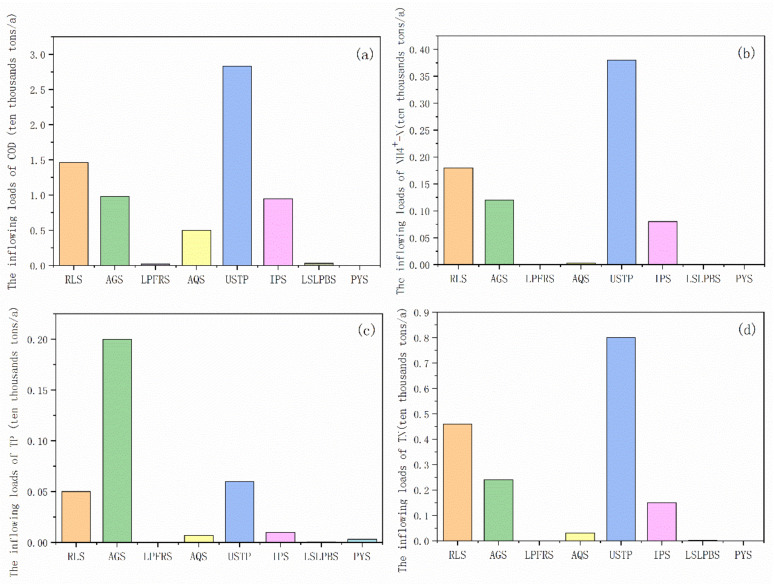
The inflowing load of chemical oxygen demand (**a**), ammonia nitrogen (**b**), total phosphorus (**c**) and total nitrogen (**d**) from different pollutants sources. The y-axis represents the pollutant loads from the RLS (rural living source), AGS (Agricultural source), LPFRS (Livestock and free range poultry sources), AQS (Aquaculture source), USTP (Urban sewage treatment plant), IPS (Industrial point source), LSLPBS (Large-scale livestock and poultry breeding source), PYS (Phosphogypsum yard source).

According to SWAT model results, the pollution loads of all pollutants are shown in Figure 5. The total inflowing loads of COD, TN, TP, and N-NH4^+^ were 67,700 tons, 16,800 tons, 3300 tons, and 7600 tons, respectively, for the Tuojiang River Basin in 2016.

Similar to the total pollutants’ emissions, the urban sewage treatment plant point source makes the largest contribution to COD, TN, and N-NH4^+^ inflowing load, with the total inflowing loads of 28,300 tons, 8000 tons, and 3800 tons, respectively. Agricultural sources still act as the dominant factor for TP, with contribution loads of 2000 tons. In particular, the Phosphogypsum yard source discharged 0.91% of the TP. Phosphorus (P) is one of the most important mineral nutrients in agricultural systems, and is generally the most limiting nutrient for plant production [43]. With the deepening of water pollution control in the Tuojiang River Basin, the problem of TP from agricultural pollution has gradually become prominent [23].

Urban sewage treatment plant, rural living source, agricultural source and industrial point source are the main sources of COD, TN and N-NH4^+^. For COD, the urban sewage treatment plant point source has the largest contribution rate, with a contribution rate of 41.80%, followed by rural living sources, agricultural sources and industrial point source, with contribution rates 21.57%, 14.48% and 14.03% respectively. The TN and N-NH4^+^ shows same characteristics as COD, the urban sewage treatment plants produced 47.55% TN and 49.76% N-NH4^+^. Similar results were reported by previous studies, which described that with field investigations and observations data, medium-sized and small rivers have been seriously polluted by industrial and domestic sewage, animal manures, chemical fertilizers in farmland, and polluted sediments.

In addition to the main sources, the percentage contributions of other pollution sources also deserve attention. For example, although the contribution of aquaculture was low, the worsening eutrophication and the resulting ‘red tide’ [44,45] have become increasingly serious as the output of products continued to rise (the output of aquatic products increased by more than 40 times) [10]. Accumulation of nutrients in aquatic systems can have negative impacts on water quality [46].

#### 3.2.2. Monthly and Seasonal Pollution Loads of Different Pollution Sources

**Figure 6:**
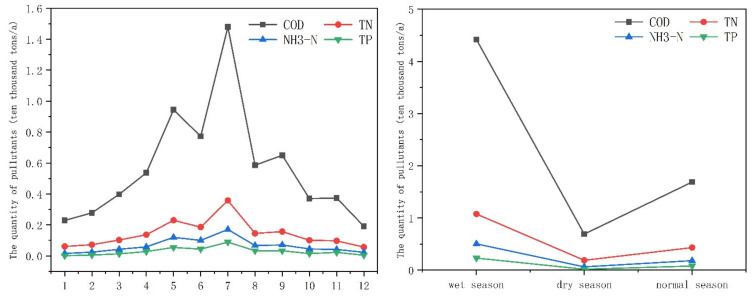
Trend of total pollution load in different water periods and months.

To better understand the temporal characteristics of the pollution loads, an analysis of the contributions in different months and water periods was carried out. According to the characteristics of rainfall and climate in the Tuojiang River basin, the wet season is from May to September, the dry season is from December to March, and the remaining months are normal. The monthly and water period variation characteristics of four pollutants entering the Tuojiang are shown in Figure 6.

On the whole, the variation with months and water periods of four pollutants loads tends to be consistent. The pollutant load of inflow into the river in the first half of the year is slightly higher than the second half. Affected by rainfall and runoff during the rainy season, the four pollutants’ load from May to September was higher than that of the remaining months, reaching the maximum value in July and the minimum value in December. The water periods characteristics of four pollutants loads are wet season > normal season > dry season, and there was an obvious decline from the wet season to the dry season. The amount of pollutants flowing into the river during the normal season is slightly higher than that in the dry season, but there is still a significant difference compared with the wet season. As the pollution load reductions during the wet season were larger than that during other seasons, the wet season was identified to be a critical period of water pollution control [47].

During the rainy season (range from May to September), the runoff carries a large amount of pollutants into the river, and rainfall accelerates the rate of pollutants entering the river, which greatly increases the pollution load in the river [48]. After July, the frequency of rainfall and the amount of precipitation gradually decreased, and pollutants entering the river decreased month by month.

**Figure 7:**
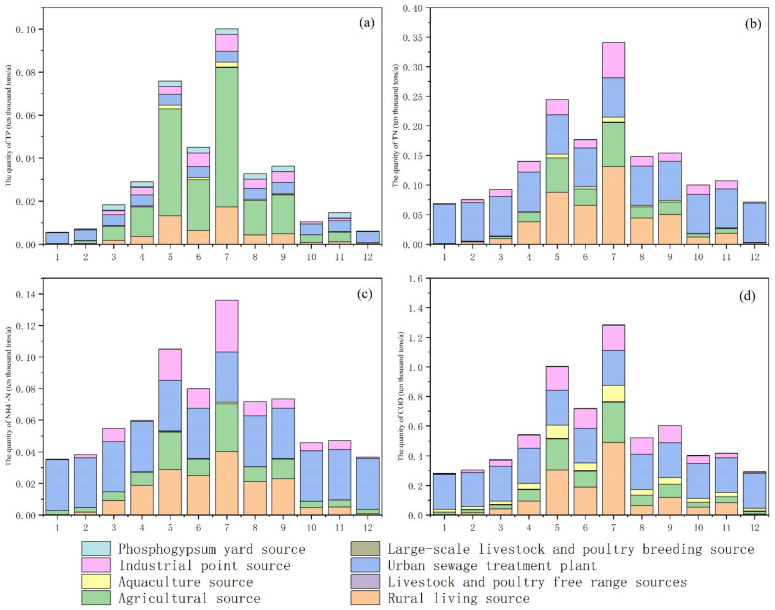
The contribution of different pollution sources to chemical oxygen demand (**a**), total nitrogen (**b**), total phosphorus (**c**) and ammonia nitrogen (**d**) in different months.

Figure 7 shows the variation characteristics of the contribution of different pollution sources to the pollution load. Urban sewage treatment plants are the largest sources of COD, TN, and N-NH4^+^, and their emissions are not affected by month and rainfall variation. They will not be affected by rain and other factors due to its fixed sewage conduit during the process of entering the river. From May to September, the months with the heaviest rainfall, the contribution of urban sewage treatment plants and rural living sources rises significantly, which is related to the people’s living characteristics. For example, the water consumption and number of baths in summer will increase. In contrast to the above three pollutants, the biggest pollution source of TP is agricultural planting sources. Many studies also indicated that agricultural production is the major source of TP [10,18]. As non-point sources, the emissions from agricultural sources are significantly affected by rainfall variation and human farming activities.

### 3.3. Spatial Characteristics Pollution Loads

#### 3.3.1. Pollution Loads of Different Administrative Regions

Since the types and contribution rate of pollution sources are related to the urbanization level, and the socioeconomic structure, agricultural practices, and rural conditions are difficult to change in a short time [49,50], it is necessary to identify the pollution load of different administrative regions scale [51].

**Figure 8:**
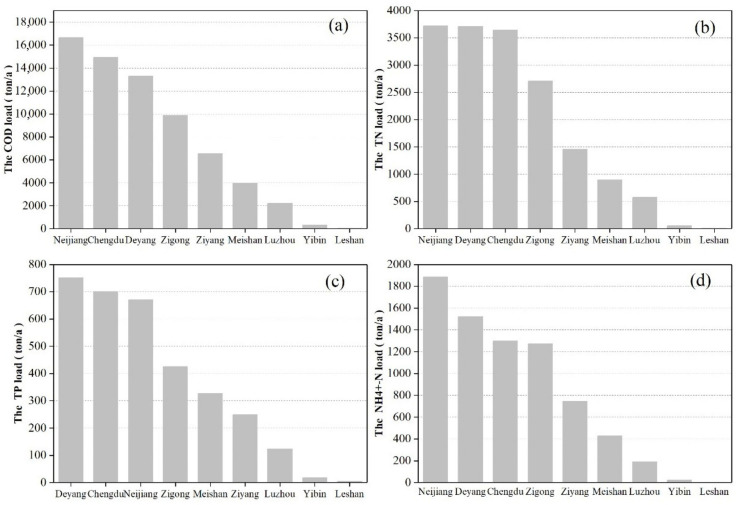
Pollutant load of chemical oxygen demand (**a**), total nitrogen (**b**), total phosphorus (**c**) and ammonia nitrogen (**d**) in different cities.

The pollution loads results of different cities are shown in Figure 8. As shown in Figure 8, Neijiang, Deyang, Chengdu and Zigong were the main producers of pollution load, and their COD, TN, TP and N-NH4^+^ loads exceeded 9000 tons, 2500 tons, 400 tons and 1200 tons, respectively. Neijiang City is the largest contributor of COD, TN, and N-NH4^+^ pollution load. 73.88% of the total area of Neijiang City is the Tuojiang River Basin, with an area of 5185 km^2^. Therefore, industrial production, agricultural production, and residential use of Neijiang are heavily reliant upon the Tuojiang River. At the same time, a large amount of above sewage is discharged to the Tuojiang River which leads to Neijiang became the primary cause of pollution [52,53]. Deyang City is the largest contributor of TP pollution load. Deyang is located in the upper reaches of the Tuojiang River Basin, the important phosphorus chemical industry base in China. Affected by this, the phosphorus pollution of the upper reaches of the Tuojiang River is more prominent, and Deyang is the area most seriously affected by phosphate rock and phosphate chemical industry in the upper reaches of the Tuojiang River [54].

According to the environmental statistics annual report data, there were 1731 industrial enterprises included in the key survey in the Tuojiang River Basin in 2016, including 495 in Chengdu, 417 in Zigong, 287 in Deyang, 179 in Neijiang, 178 in Luzhou, 147 in Ziyang, and 28 in Meishan. The main pollutant sources are regions with more industrial enterprises and sewage treatment plants, which lead to the Neijiang, Deyang, Chengdu and Zigong become the four biggest source of pollution [30].

**Figure 9:**
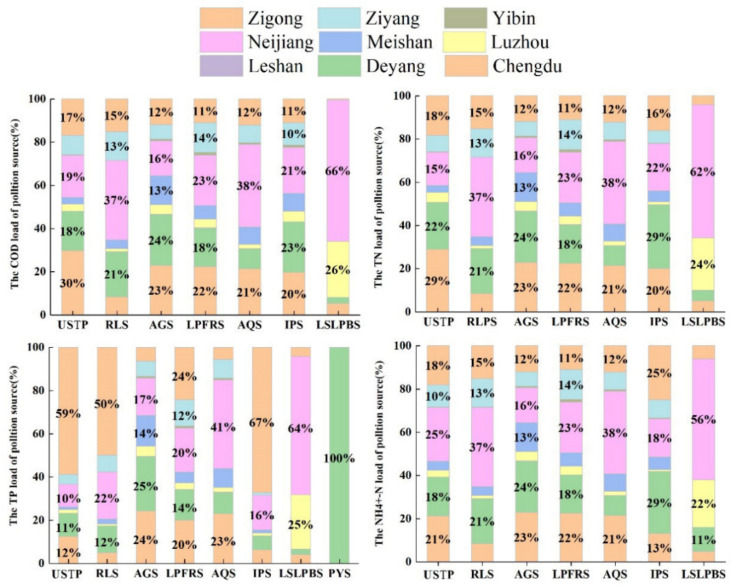
The contribution ratio of different cities to different pollution sources. The y-axis represents the pollutant loads from the USTP (Urban sewage treatment plant), RLS (rural living source), AGS (Agricultural source), LPFRS (Livestock and free range poultry sources), AQS (Aquaculture source), IPS (Industrial point source), LSLPBS (Large-scale livestock and poultry breeding source) and PYS (Phosphogypsum yard source).

On the basis of grasping the pollution load of different cities, we further explored the contribution rates of the above nine cities to different pollution sources, and the results are shown in Figure 9. The contribution rate of the nine cities to different pollution sources is related to their own urbanization level and functional positioning. Neijiang is the main source of pollution loads from large-scale breeding, aquaculture and rural life. This is because Neijiang has a rural population of 3,046,300 and an area of cultivated land of 163,100 hectares [28,55]. It has a large-scale commercial grain base, a raw pork production base, and the main production area of Sichuan grain and economic crops nationwide. The main sources of pollution from urban sewage treatment plants are Deyang, Chengdu, and Zigong, which are located in the upper and lower reaches, with a high level of urbanization and dense population, so a large amount of domestic sewage is discharged into the sewage treatment plant. Deyang is the biggest contributor to pollution from industrial enterprises, because Deyang is an important phosphorous chemical base, and it undertakes the construction of important industrial projects in China [27,54].

#### 3.3.2. Pollution Load of Different Sub-Watershed

**Figure 10:**
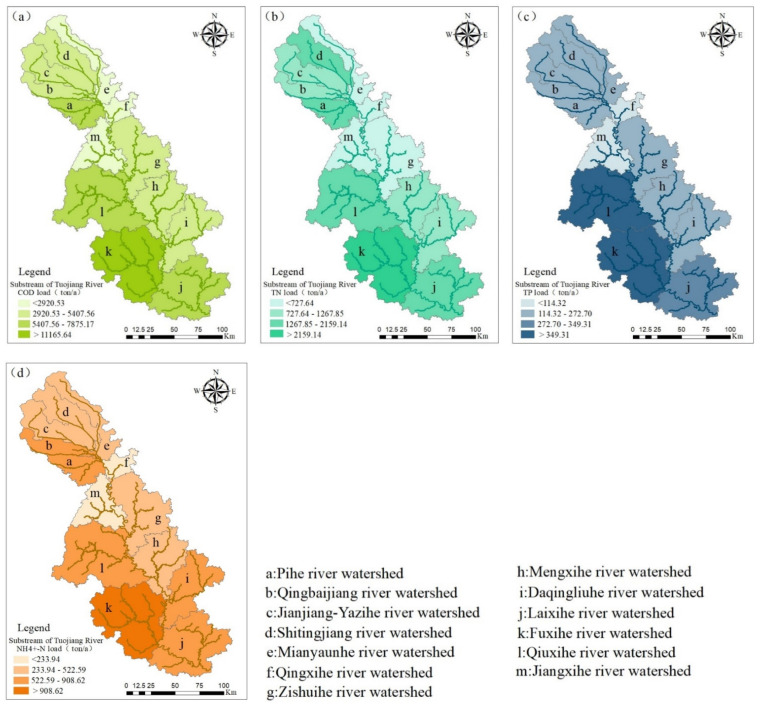
The chemical oxygen demand (**a**), total nitrogen (**b**), total phosphorus (**c**) and ammonia nitrogen (**d**) pollution loads of different sub-basins, where a~m represent the Pihe river watershed, Qingbaijiang river watershed, Jianjiang-Yazihe river watershed, Shitingjiang river watershed, Mianyuanhe river watershed, Qingxihe river watershed, Zishuihe river watershed, Mengxihe river watershed, Daqingliuhe river watershed, Laixihe river watershed, Fuxihe river watershed, Qiuxihe river watershed, Jiangxihe river watershed respectively.

There are huge differences in the water quality of different tributaries, so we further studied the pollution loads of different sub-watershed as shown in Figure 10.

From the figure, we can conclude that the sub-basins of Fuxihe River (k) and Qiuxihe River (l) are the most heavily polluted sub-basins, followed by Pihe River (a) and Laixi River (j). The hydrological characteristics of the above three sub-basins are relatively complex, except for the Pihe River. Common to three sub-basins is that more than four small tributaries flow into their catchment areas, and the pollutants carried by them may be the major reason for the load of the sub-basins. The more complex hydrologic conditions and more tributaries in the sub-basin there are, the more difficult it is to analyze the source, which leads to the increase of pollution load in the catchment area.

The Fuxi River is the first-level tributary of the Tuojiang River and the only river that passes through the central area of Zigong City, which has typical urban river pollution characteristics, i.e., the river is seriously polluted by industrial enterprises and sewage treatment plants [56]. It is responsible for nearly three million people’s domestic water in Zigong area, and its pollutant emissions far exceed its environmental capacity. In contrast to the Fuxi River, the Qiuxi River Basin involves 49 townships, mainly involving districts and counties including Renshou County of Meishan City, Yanjiang District of Ziyang City, and Zizhong County of Neijiang City, which is a typical rural river, polluted by rural living sewage and agricultural non-point sources [29,57]. Due to the low level of urbanization in towns and villages, the environmental protection infrastructure is seriously inadequate. For example, in 2016, the population of Renshou County reached more than 400,000, but there was only one sewage treatment plant. Untreated rural domestic water directly entered the river and caused serious pollution to the river. The Pihe River mainly involves seven districts and counties of Chengdu, Dujiangyan, Pixian, Xindu, Jinniu District, Qingbaijiang, Longquanyi, and Jintang. The pollution sources of Pihe are mainly distributed in Xindu and Qingbaijiang districts. Enterprises are concentrated in Qingbaijiang. The factories are concentrated in Xindu and Qingbaijiang [40].

After the construction of sluice gates on many tributaries of the Tuojiang River Basin, the flow rate of the river slowed down and a large amount of pollutants accumulated in the sediment. In addition, more sewage is directly discharged into tributaries. The combined effect of internal pollution and external release has led to the deterioration of the water quality of the tributaries of the Tuojiang River [58].

## 4. Conclusions

According to the characteristics of emission and flow into the river of different pollution sources including point sources and non-point sources, we have constructed a basin-scale pollution loads analysis method of coupling investigation method, empirical formula method and non-point source model method. This method has been applied in the Tuojiang River Basin, and total pollution emissions and total inflowing loads have been calculated. Our results demonstrated that urban sewage treatment plant contributed most to COD, TN and N-NH4^+^-N followed by rural living sources. Agricultural sources are the main pollution source of total phosphorus (TP) throughout the year. In the rainy season, precipitation significantly increases the pollution load of agricultural sources but the change of industrial point sources is very stable. Neijiang city and Deyang city contribute the most to COD, N-NH4^+^, TP and TN, and the Fuxi River subbasin and Qiuxihe River subbasin contribute the most pollutant load among the sub-basins.

The analysis of pollution sources is a difficult challenge, and there are still certain problems with our method. For example, the calculation of pollution source emissions based on the pollutant discharge coefficient method does not take into account the spatial characteristics of different sub-basins, and this method is commonly used in most current studies, but the discharge coefficient of different regions is different. In more detailed studies, different coefficient analysis should be carried out according to the characteristics of different sub-basins. On the other hand, the SWAT model is a non-point source model commonly used at home and abroad, but the model needs to be improved in the simulation of COD. However, overall, our methods and results can identify the main pollution source types and pollution areas. The technical framework of this article can provide a reference for similar river basins and can be applied to more situations, such as the targeted treatment of environmental pollution, water pollution source tracking and other targeted strategy needs.

## Figures and Tables

**Table 1:** Calculation equations of discharge coefficient method.

Pollution Sources	Formula	Symbols and Units	Remarks
Rural living sources	$G_{p}=365\times NS_{p}\times 10^{-10}$	Where *G_p_* represents some kind of pollution emission (10,000 tons/year); *N* represents Number of permanent residents (one person); *S_p_* represents discharge coefficient of pollutants (g/day·person)	The pollution loads of COD, TN, TP, and N-NH4^+^ are calculated by the same formula.
Agricultural source	$P=\sum (A_{N}F_{N}+A_{P}F_{P})/10^{4}$	Where *P* represents a load of a certain pollutant (10,000 tons/year); *A_N_*, *A_P_* represents the amount of Nitrogen and Phosphorus fertilizer applied in farmland (Tons/Year); *F_N_*, *F_P_* represents loss Coefficient of Nitrogen and Phosphate Fertilizer (%)	
Livestock and poultry breeding source	$M=\sum C_{i}P_{i}\times 365/10^{7}$	Where *M* represents the discharge of certain pollutants (10,000 tons/year); *C_i_* represents the total number of animals of type *i* (one head); *P_i_* represents type *i* animal emission factor (kg/head (only)····day)	
Aquaculture source	$M_{N}=(C\times N_{f}-N_{b})\times 10^{3}$ $M_{P}=(C\times P_{f}-P_{b})\times 10^{3}$	*MN*, *MP* Represents nitrogen load and phosphorus load (kg/ton) respectively; *C* is the bait coefficient; *N_f_*, *P_f_* represents the percentage of nitrogen and phosphorus in the bait respectively; *N_b_*, *P_b_* represents the content percentage of nitrogen and phosphorus in cultured fishery products respectively	Applicable to the calculation of TN and TP pollution load

**Table 2:** Export coefficients of pollution sources.

Type		Waste Water (L/Day)	COD	TN	TP	NH3-N
Rural living sources g/day·person	Chengdu	130.00	52.00	10.10	0.97	7.10
	Zigong	120.00	50.00	10.20	0.91	7.30
	Luzhou	130.00	52.00	10.10	0.97	7.10
	Deyang	140.00	57.00	10.90	0.97	7.70
	Neijiang	150.00	66.00	10.60	1.07	7.30
	Leshan	140.00	57.00	10.90	0.97	7.70
	Meishan	125.00	50.00	10.70	0.97	7.80
	Yibin	125.00	50.00	10.70	0.97	7.80
	Ziyang	130.00	52.00	10.10	0.97	7.10
Agricultural source %	Nitrogen fertilizer			1.85	1.55	1.35
	Phosphorus fertilizer			1.85	1.55	1.35
Livestock and poultry breeding source kg/day	Pig	4.42	0.40	0.02	0.00	0.01
	Cow	20.42	2.24	0.10	0.01	0.03
Aquaculture source g/kg	Fish		40.76	3.58	0.70	1.19

**Table 3:** Multisource data for the SWAT model.

Data Name	Datatype	Accuracy	Sources
DEM	.tif	30 m	Chinese Academy of Sciences mirror site
Land-use type map	.shp	30 m	Sichuan Academy of Environmental Sciences
The soil type of map	.shp	1:1,000,000	Harmonized World Soil Database version (HWSD)
Meteorological data	.txt	-	China Meteorological Science Data Center
Hydrological and water quality monitoring data; agricultural management farming system; point source pollution data	.xls	-	Sichuan Provincial Ecological Environment Monitoring Station

**Table 4:** The discharge of pollutants of different sources.

Pollution Source	Pollution Type	The Quantity of Pollutant Discharge (10,000 Ton/Year)			
		COD	TN	TP	N-NH4^+^
NPS	Rural living source	13.67	3.64	0.26	1.82
	Agricultural source	10.06	2.01	1.68	1.47
	Livestock and free range poultry sources	0.19	0.01	0	0.01
	Aquaculture source	9.76	0.86	0.17	0.14
PS	Urban sewage treatment plant	19.84	3.93	0.35	2.57
	Industrial point source	0.95	0.15	0.01	0.08
	Large-scale livestock and poultry breeding source	0.031	0.002	0.0005	0.0004
	Phosphogypsum yard source	0	0	0.003	0
Total		54.5	10.60	2.47	6.09

## Data Availability

The data presented in this study are available upon request from the corresponding author. The data are not publicly available because of privacy concerns.
